# Effects of rosuvastatin/ezetimibe on senescence of CD8+ T-cell in type 2 diabetic patients with hypercholesterolemia: A study protocol

**DOI:** 10.1097/MD.0000000000031691

**Published:** 2022-11-25

**Authors:** Sang Hyeon Ju, Bon Jeong Ku

**Affiliations:** a Department of Internal Medicine, Chungnam National University Hospital, Daejeon, Republic of Korea; b Department of Internal Medicine, Chungnam National University School of Medicine, Daejeon, Republic of Korea.

**Keywords:** ezetimibe, senescent CD8 + T cells, statin, type 2 diabetes mellitus

## Abstract

A decade ago, systemic inflammation became widely recognized as an etiology of type 2 diabetes mellitus (T2DM) and complications thereof. Senescent CD8 + T cells of T2DM patients exhibit increased secretion of pro-inflammatory cytokines and enhanced expression of cytotoxic molecules, contributing to systemic inflammation. Recently, many anti-inflammatory roles played by statins and ezetimibe (cholesterol-lowering drugs) have been reported. We will explore the effects of statin/ezetimibe therapy on CD8 + T cell senescence in patients with T2DM and hypercholesterolemia.

**Methods:**

This 2-group, parallel, randomized, controlled clinical trial will recruit 108 subjects with T2DM and low-density lipoprotein-cholesterol (LDL-C) levels ≥100 mg/dL and randomly assign them to rosuvastatin/ezetimibe and rosuvastatin groups at a 1:1 ratio. Blood samples will be drawn at baseline and after 12 weeks of medication. The primary outcomes will be the LDL-C-lowering effects after 12 weeks. The secondary outcomes will be changes in the senescent (CD28 − CD57+) CD8 + T cell proportions; the levels of circulating pro-inflammatory cytokines, cytotoxic molecules, interleukin-1, transforming growth factor-β, fasting glucose, and HbA1c; and biochemical indices of kidney, liver, and muscle function. Symptoms and signs of predictable adverse events (myopathy and hepatitis) will be routinely monitored.

**Discussion:**

We will evaluate the effects of statin/ezetimibe on CD8 + T cell senescence. Statin/ezetimibe may exert a beneficial immunomodulatory effect.

## 1. Introduction

### 1.1. Background

Type 2 diabetes mellitus (T2DM) is a metabolic disorder characterized by dysregulation of carbohydrate, lipid, and protein metabolism attributable to impaired insulin secretion, insulin resistance, or both.^[1]^ T2DM patients with long-standing hyperglycemia are at risk of both microvascular and macrovascular complications, elevating cardiovascular mortality^[2,3]^ attributable to the accumulation of advanced glycation end-products, activation of the polyol and aldose reductase pathways, and increased oxidative stress.^[4,5]^ Recently, chronic low-grade inflammation triggered by circulating inflammatory cytokines released from activated immune cells including macrophages, T cells, B cells, and NK cells has been suggested to initiate both diabetes and the complications thereof.^[6–9]^

CD8 + T cells (cytotoxic T cells) mediating adaptive immunity kill cancer cells, infected cells, and cells otherwise damaged.^[10]^ CD8 + T cell senescence contributes to the development of chronic low-grade inflammation via the secretion of pro-inflammatory cytokines and expression of cytotoxic molecules, including granzyme B and perforin.^[11,12]^ Recent studies found that the proportions of senescent CD8 + T cells were increased in T2DM patients and that pro-inflammatory cytokines and cytotoxic molecules secreted by such cells induced chronic low-grade inflammation^[13,14]^ associated with the development of cardiovascular complications.^[13,15,16]^ Inhibition of such senescence might prevent cardiovascular disease in T2DM patients.

Statins are selective competitive inhibitors of hydroxymethylglutaryl-CoA reductase (the rate-limiting enzyme in cholesterol biosynthesis). Statins not only lower cholesterol levels but also improve endothelial function, inhibit vascular inflammation, reduce oxidative stress, and stabilize plaque.^[3,17–19]^ Given such immunomodulatory actions, statins have been used as adjuvants to enhance antigen presentation and to inhibit cholesterol-induced CD8 + T cell exhaustion in tumor microenvironments.^[20–22]^ However, any effect of statins on CD8 + T cell senescence in T2DM patients remains unknown.

Ezetimibe inhibits intestinal cholesterol absorption, thus reducing serum cholesterol levels; statin/ezetimibe combination therapy more effectively prevents cardiovascular disease than does statin monotherapy.^[23]^ Ezetimibe affects endothelial function^[24,25]^; any effect of ezetimibe on CD8 + T cell senescence remains unknown. Therefore, we will investigate the effect of statin/ezetimibe therapy on such senescence in patients with T2DM and hypercholesterolemia.

### 1.2. Objectives

1. To compare the low-density lipoprotein-cholesterol (LDL-C)-lowering effects of 12 weeks of rosuvastatin with or without ezetimibe in T2DM patients with LDL-C levels ≥ 100 mg/dL.
2. To explore the effects of 12 weeks of rosuvastatin with or without ezetimibe on senescent CD8 + T cell expressions.

## 2. Methods

### 2.1. Trial design

This will be a 2-group, parallel, randomized control study using either rosuvastatin with ezetimibe (experimental group) or rosuvastatin only (control group). Participants (n = 108) will be randomly assigned to the experimental (n = 54) and control (n = 54) groups.

### 2.2. Ethics statement

The research protocol adheres to the Declaration of Helsinki and the Ethical Guidelines for Clinical Research and was approved by the Ethics Committee of the Institutional Review Board of Chungnam National University Hospital. Informed consent was provided by all subjects upon enrollment (IRB file no. CNUH 2018-10-030); all have been told that they may withdraw at any time. The protocol conforms to the Standard Protocol Items: Recommendations for Interventional Trials (SPIRIT) 2013 statement^[26]^ and the revised CONSORT guidelines.^[27]^ Direct access to the source data will be provided during and after the study to a monitoring body, for research ethics committee/institutional review board review, and for inspections by the regulatory authority. The protocol has been registered with the Clinical Research Information Service (no. KCT0006625) and will be updated during the study.

### 2.3. Participants and eligibility criteria

Of patients visiting the Diabetes Center at Chungnam National University Hospital, those diagnosed with T2DM and an LDL-C level ≥ 100 mg/dL will be included.

#### 2.3.1. Inclusion criteria.

The inclusion criteria are:

1. Males or females > 18 years of age.
2. A diagnosis of T2DM using the criteria of the Standards of Medical Care in Diabetes (2022) of the American Diabetes Association.^[28]^
3. An LDL-C level ≥ 100 mg/dL.

#### 2.3.2. Exclusion criteria.

The exclusion criteria are:

1. Patients on lipid-lowering therapies (statins, ezetimibe, fibrates, nicotinic acid, bile acid sequestrants, and PCSK9 inhibitors).
2. Patients on postmenopausal hormone replacement therapy.
3. Patients with an aspartate aminotransferase (AST) or an alanine aminotransferase (ALT) level > 2.5-fold the upper normal limits.
4. Patients with creatine kinase (CK) levels > 3-fold the upper normal limit.
5. Patients diagnosed with or treated for malignant tumors within the past 5 years.
6. Patients with uncontrolled or poorly controlled hypothyroidism (thyroid-stimulating hormone levels > 1.5-fold the upper normal limit).
7. Patients hypersensitive to rosuvastatin, ezetimibe, or other statins.

### 2.4. Study setting

In this single-center study, participants will be recruited and treated at the Diabetes Center of Chungnam National University Hospital (a tertiary hospital that trains medical staff). Data will be collected at baseline and at the end of the study.

### 2.5. Interventions

**Figure 1.**
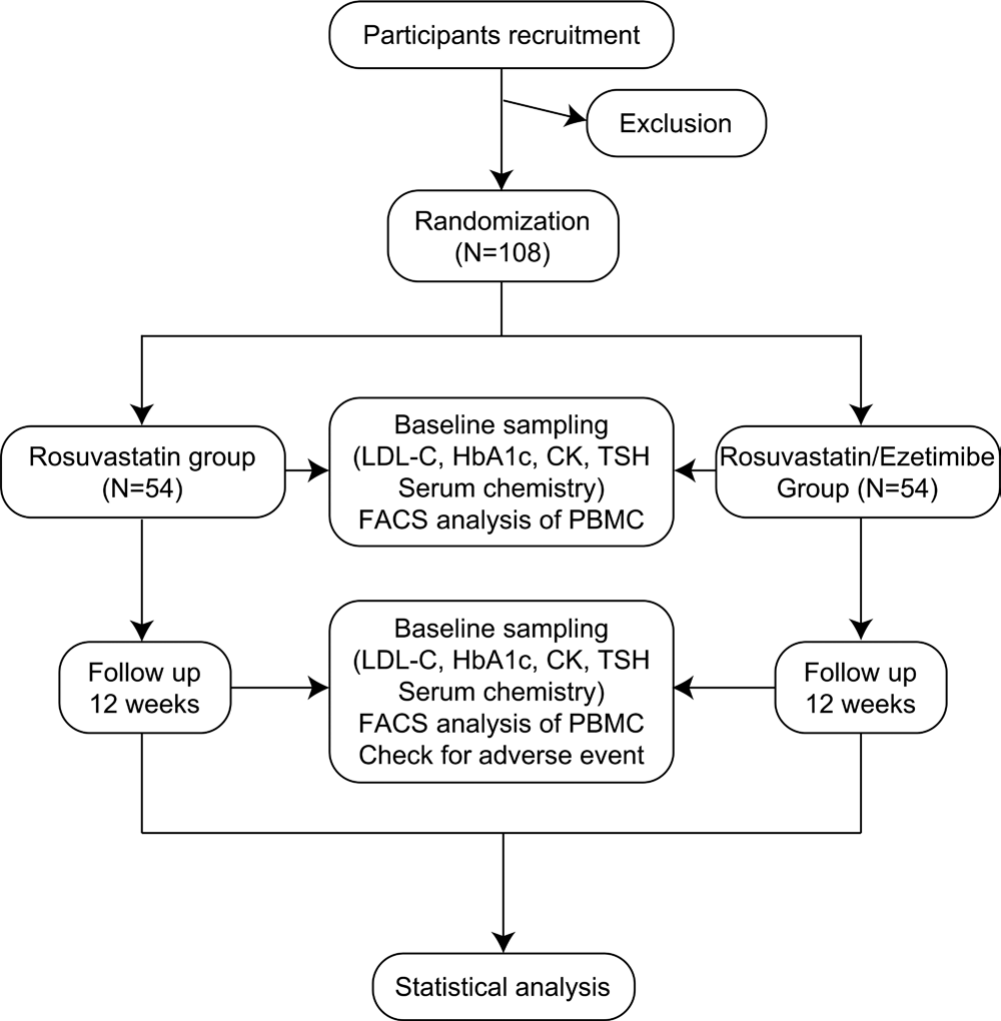
Study scheme of the proposed clinical trial. CK, creatine kinase, FACS = Fluorescence-Activated Cell Sorting, LDL-C = low-density lipoprotein cholesterol, TSH, thyroid-stimulating hormone.

Subjects who meet the eligibility criteria will be randomly assigned to the rosuvastatin/ezetimibe or rosuvastatin group (Fig. 1). Both groups will receive routine diabetes care and treatment during the 12 weeks of the study. The rosuvastatin/ezetimibe group will be prescribed tablets of rosuvastatin (5 mg) and ezetimibe (10 mg), and the rosuvastatin group tablets of rosuvastatin (5 mg), for 12 weeks. Both groups will take pills once daily 30 min after breakfast. Blood samples will be drawn before and at the end of the study to derive the lipid profile (LDL-C, high-density lipoprotein-cholesterol, triglyceride, and total cholesterol levels), the HbA1c level, the senescent CD8 + T cell number, and other parameters.

### 2.6. Outcome measurements

#### 2.6.1. Primary outcome.

The primary outcome is the LDL-C-lowering effect in the 2 groups and a comparison thereof. At baseline and at the end of the study, peripheral blood will be collected into serum separation tubes (SST tubes) and LDL-C levels determined using the colorimetric enzymatic technique of the Cobas 6000 analyzer (Roche Diagnostics, Basel, Switzerland).

#### 2.6.2. Secondary outcomes.

1. Changes in the proportions of senescent CD8 + T cells (CD28 − CD57 + CD8 + T cells) 12 weeks from baseline and a comparison between the 2 groups.
2. Changes in the proportions of senescent CD8 + T cells (CD28 − CD57 + CD8 + T cells) 12 weeks from baseline relative to the LDL-C changes and a comparison between the 2 groups.
3. The levels of pro-inflammatory cytokines (interferon [IFN]-*γ*, tumor necrosis factor [TNF]-*α*, and interleukin [IL]-17A) expressed by senescent CD8 + T cells (CD28 − CD57 + CD8 + T cells) at 12 weeks compared to baseline and a comparison between the 2 groups.
4. The levels of cytotoxic granzyme B and perforin expressed by senescent CD8 + T cells (CD28 − CD57 + CD8 + T cells) at 12 weeks compared to baseline and a comparison between the 2 groups.
5. The changes in serum IL-1 and transforming growth factor-β levels during the 12 weeks of the study evaluated using commercial ELISA kits and a comparison between the 2 groups.
6. The changes in fasting glucose and HbA1c levels during the 12 weeks of the study evaluated using commercial ELISA kits and a comparison between the 2 groups.
7. Assays of kidney function, including blood urea nitrogen and creatinine levels, and the estimated glomerular filtration rates derived using the CKD-EPI equation.
8. Serum assays of liver function (AST, ALT, total bilirubin, and albumin levels).
9. Serum CK and thyroid-stimulating hormone levels at baseline and the end of the study.

For secondary outcomes 1 to 4, peripheral blood mononuclear cells will be isolated and subjected to flow cytometric analysis as described previously by our group.^[29]^ The monoclonal antibodies will be anti-CD4-AF700, anti-CD8-PE, anti-CD8-APC, anti-CD28-APC, anti-CD57-FITC, anti-IFN-*γ*-PE-Cy7, anti-TNF-*α*-APC, anti-IL-17A-APC, anti-granzyme B-PE, and anti-perforin-APC (all from eBioscience, San Diego, CA, USA). Permeabilized cells will be stained for intracellular cytokines and cytotoxic molecules using anti-IFN-*γ*-PE-Cy7 and anti-TNF-*α*-APC. Multicolor flow cytometry will employ a BD LSRFortessa flow cytometer (BD Biosciences, San Jose, CA, USA) and the data will be analyzed with aid of FlowJo V10 software (FlowJo, LLC, Ashland, OR, USA).

#### 2.6.3. Safety.

All adverse events will be documented on a “Case Report” form. Possible adverse effects (e.g., muscle pain/weakness, headache/dizziness, sleep problems, nausea, indigestion, diarrhea, and constipation) will be routinely reviewed. Sera will be assayed for AST, ALT, and CK to reveal asymptomatic adverse effects. Events not directly related to treatment will also be recorded.

### 2.7. Sample size

The sample size was calculated based on the LDL-C-lowering rates of rosuvastatin/ezetimibe (61.60 ± 19.58%) and rosuvastatin (50.50 ± 19.50%) (unpublished data). These were entered into a size calculation website (https://clincalc.com/stats/SampleSize.aspx). For a power (1-*β*) of 0.8, an *α* = 0.05, and a 1:1 group ratio, 49 subjects were required to compare the LDL-C-lowering efficacies. After allowing 10% for follow-up loss/drop-off, we finally included 54 subjects/group.

### 2.8. Randomization and masking

Eligible subjects will be randomly assigned to either the rosuvastatin/ezetimibe or rosuvastatin group at a 1:1 ratio. A random allocation sequence will be generated using SPSS ver. 26.0 (IBM Corp., Armonk, NY, USA). Participants will be given a number by a doctor and will pick up medication at the pharmacy. Doctors and pharmacists will be blinded to the chosen drug. After collection of 12-week data, blinding will be lifted.

### 2.9. Data analysis

Data from baseline and at 12 weeks will be collected using a standardized data sheet; all patients will be anonymized. The paired t-test will be used to compare within-group differences between baseline and 12 weeks. The unpaired t-test will be employed to compare between-group changes. Statistical significance will be defined as *P* < .05. Statistical analyses will be performed with the aid of SPSS ver. 26.0 and figures drawn using GraphPad Prism ver. 9.4.1 (GraphPad Software Inc., San Diego, CA) and OriginPro 2021 (OriginLab Corp., Northampton, MA).

## 3. Discussion

We will evaluate the effect of statin/ezetimibe therapy on CD8 + T cell senescence in patients with T2DM and hypercholesterolemia. The pathophysiology of T2DM was described as an “ominous octet” in 2009^[30]^; this became a “decadent decuplet” in 2015 after the inclusion of vascular insulin resistance and inflammation.^[1]^ Accumulating evidence suggests that systemic inflammation contributes to both insulin resistance and pancreatic β-cell dysfunction. Glucotoxicity- and lipotoxicity-induced local production of cytokines and chemokines in pancreatic β-cells and insulin-sensitive tissues including adipose tissue and liver initiate the inflammation associated with T2DM.^[31]^ Pro-inflammatory cytokines (IL-1β, IL-6, and TNF-α) and chemokines released from tissues may recruit and regulate the differentiation and activation of immune cells, triggering the progression of local inflammation.^[9,32]^ Importantly, senescent (CD28 − CD57+) CD8 + T cells are highly inflammatory. Such cells secrete cytotoxic mediators and bypass antigen specificity; the numbers of such cells are increased in T2DM patients.^[13,33]^ We previously showed that senescent T cells increased systemic inflammation and rendered hepatic glucose homeostasis abnormal.^[29]^

Traditionally, statins are thought to induce insulin resistance, especially in patients with baseline risk factors for T2DM using high-intensity statin therapy.^[34]^ However, in recent years, statins have been shown to improve endothelial function, inhibit vascular inflammation, reduce oxidative stress, and stabilize plaque.^[3,17–19]^ Cholesterol- and statin-mediated immunomodulation was evident under specific conditions; increased cholesterol levels in tumor microenvironments induced CD8 + T cell exhaustion,^[21]^ atorvastatin reduced T cell activation and exhaustion in HIV-infected combination antiretroviral therapy-treated patients,^[35]^ and rosuvastatin induced apoptosis in CD4 + CD28null T cells in patients with acute coronary syndrome.^[36]^ The mechanisms connecting statins to T cell exhaustion or senescence remain unclear. Statin immunomodulation may involve a telomerase reverse transcriptase (TERT)-mediated mechanism,^[37]^ low-density lipoprotein receptor-mediated T cell receptor recycling and signaling,^[38]^ expression of Kruppel-like factor 2,^[39]^ downregulation of a co-inhibitory receptor by atorvastatin,^[40]^ and/or p53 isoform (Δ133p53α)-induced CD28 expression.^[41]^

In brief, prior studies have suggested that statins play immunomodulatory roles. We will explore whether statins affect CD8 + T cell senescence in T2DM patients. The trial has certain limitations. First, there is no placebo control. Second, all subjects will be recruited from the diabetes center of a single tertiary hospital; any generalization of the results will require caution.

## Acknowledgments

The authors thank all participants in this trial.

## Authors contribution

**Conceptualization:** Bon Jeong Ku.

**Data curation:** Sang-Hyeon Ju.

**Formal analysis:** Sang-Hyeon Ju.

**Funding acquisition:** Bon Jeong Ku.

**Investigation:** Sang-Hyeon Ju.

**Methodology:** Bon Jeong Ku.

**Project administration:** Bon Jeong Ku.

**Resources:** Bon Jeong Ku.

**Software:** Sang-Hyeon Ju.

**Supervision:** Bon Jeong Ku.

**Validation:** Bon Jeong Ku.

**Visualization:** Sang-Hyeon Ju.

**Writing—original draft:** Sang-Hyeon Ju.

**Writing—review & editing:** Bon Jeong Ku.
